# Gallic acid abates cadmium chloride toxicity via alteration of neurotransmitters and modulation of inflammatory markers in Wistar rats

**DOI:** 10.1038/s41598-023-28893-6

**Published:** 2023-01-28

**Authors:** Oluwafemi Adeleke Ojo, Damilare Emmanuel Rotimi, Adebola Busola Ojo, Akingbolabo Daniel Ogunlakin, Basiru Olaitan Ajiboye

**Affiliations:** 1 Phytomedicine, Molecular Toxicology, and Computational Biochemistry Research Laboratory (PMTCB-RL), Department of Biochemistry, Bowen University, Iwo, 232101 Nigeria; 2 Department of Biochemistry, Landmark University, Omu-Aran, Nigeria; 3 Department of Biochemistry, Ekiti State University, Ado-Ekiti, Nigeria; 4 Department of Biochemistry, Federal University Oye-Ekiti, Oye-Ekiti, Nigeria

**Keywords:** Biochemistry, Neuroscience

## Abstract

Cadmium is a highly neurotoxic heavy metal that disrupts membranes and causes oxidative stress in the brain. The study aimed to investigate the neuroprotective effect of gallic acid on oxidative damage in the brains of Wistar rats exposed to cadmium chloride (CdCl_2_). Male Wistar rats were divided into four groups of five rats each. Group 1 was administered distilled water only throughout the study. Throughout the study, Group 2 received CdCl_2_ alone (5 mg/kg b.w./day), Group 3 received gallic acid (20 mg/kg b.w./day), and Group 4 received CdCl_2_ + gallic acid (20 mg/kg). Treatments were oral with distilled water as a vehicle. The study lasted 21 days. In the brain, the activities of cholinesterase and antioxidant enzymes were evaluated, as well as the levels of reduced glutathione, malondialdehyde, neurotransmitters, Na+/K+ ATPase, myeloperoxidase activity, nitric oxide, and interleukin-6. CdCl_2_-induced brain impairments in experimental animals and gallic acid prevents the following CdCl_2_-induced activities: inhibition of acetylcholinesterase (AChE) and butyrylcholinesterase (BChE), elevated neurotransmitters (serotonin and dopamine), decreased antioxidant enzymes (superoxide dismutase, catalase), decreased glutathione, Na+/K+ ATPases, and increased MDA and neuroinflammatory markers (myeloperoxidase (MPO), nitric oxide, and interleukin-6 in the brain of experimental rats exposed to CdCl_2_ (*p* < 0.05). Taken together, the neuroprotective effects of gallic acid on CdCl_2_-induced toxicity in the brains of rats suggest its potent antioxidant and neurotherapeutic properties.

## Introduction

Cadmium (Cd) is a toxic heavy metal that has a negative impact on human and animal cellular and metabolic systems. Cd levels in brain tissue are always high in rats that have been intoxicated with the metal (at dosage roughly 700 times higher than the control values). Cadmium salts is one of the most toxic pollutants found in the environment. Because Cd is not broken down, the danger of human exposure to Cd via food chain pollution is constantly increasing^1^. Exposure to cadmium occurs primarily through foods such as cereals and vegetables, implying that exposure is constant. The rise in Cd contamination is a major public health concern around the world^2^. Long-term exposure to Cd is well known to cause toxic side effects in numerous organ systems, like the cardiovascular, brain, and immune/hemopoietic^3,4^. Cd has the potential to cause neurotoxicity, resulting in an extensive range of clinical entities such as neurological disturbances and changes in normal brain neurochemistry^5^. Cd has been shown to promote lipid peroxidation (LPO) by increasing free radicals^6^. Cd toxicity has been linked to the production of reactive oxygen species (ROS) and the depletion of antiradicals^7^. The capability of Cd to cause oxidative stress in brain cells has been described as ROS generation after Cd intoxication with mitochondrial sites, resulting in the breakdown of mitochondrial potential and a subsequent decrease in intracellular glutathione levels^8^. Due to its high rate of oxygen utilization, abundance of polyunsaturated fatty acids, weak antioxidant defense, and high concentration of transition metals like copper and iron in some areas, brain tissue is highly susceptible to lipid peroxidation^9^. Loss of membrane-bound ATPase activity may occur as a result of the brain membranes' increased sensitivity to LPO^10^. Lipid-dependent membrane-bound enzymes known as ATPases play a role in active transport, preservation of cell homeostasis, and neurotransmission^11^. Cadmium modifies the activities of oxidative stress-neutralizing enzymes, causing disruptions in brain metabolism and contributing to cadmium’s neurotoxic effect. Cadmium causes changes in the structural integrity of lipids and has indirect effects on membrane-bound enzymes by increasing the production of free radicals in the brain and interfering with the antioxidant defense system^12^. The ability of Cd to cross the blood–brain barrier may explain its accumulation in brain tissue^13^. Reactive oxygen species (ROS) production is a normal process in metabolism. Excessive ROS production, not compensated by antioxidants, cause oxidative stress^14^. Presently, a broad investigation is being conducted to assess the preventive effects of many natural antioxidants on metal-induced toxicities^15,16^. Antioxidants are gaining increasing attention in the treatment of diseases related to oxidative stress as well as being probable therapeutic agents for a variety of disorders. Antioxidants can mitigate the cadmium-induced reduction in ATPase activity and the elevation of oxidative injury^17^.

One of the well-studied antioxidant agents is gallic acid. Gallic acid is a common plant metabolite that has the structure of trihydroxybenzoic acid and several hydrogen atoms in its phenolic structure that easily delocalize free radicals^18,19^. Its structure explains why it has strong antioxidant properties, indicating that it can protect tissues and organs from oxidative stress^18,20^. Gallic acid has previously been reported to be a common component of various foods and herbal drugs^21^. GA protects the brain by increasing antioxidant enzymes and decreasing inflammation^22,23^. Gallic acid can reduce the incidence of inflammation-related diseases, including cancer^24,25^, cardiovascular disease^26,27^, liver disease^28,29^, inflammation^30,31^, and neurodegenerative diseases. Gallic acid can inhibit the inflammatory process by eliminating superoxide anions, inhibiting the release and activity of myeloperoxidase (MPO), and possibly participating in the accumulation of active NADPH-oxidase^31^. This suggests that gallic acid is a promising chemical agent with a variety of chemotherapeutic properties. Given gallic acid’s antioxidant and anti-inflammatory properties, it is worth considering gallic acid as a potential preventive agent against cadmium-induced oxidative stress in the brain. The purpose of the study was to examine the protective role of gallic acid against oxidative damage caused by CdCl_2_ in the brains of male Wistar rats. As a result, we investigated the effects of gallic acid on the activities of AChE, BChE, and Na+/K+ ATPase. Furthermore, we examine the protective abilities of gallic acid on antioxidant enzymes (catalase and SOD) as well as GSH levels, markers of oxidative stress (TBA Reactive Substances (TBARS)), neurotransmitters (serotonin and dopamine levels), and inflammatory markers (MPO, nitric oxide (NO), and IL-6) in the brains of rats treated with CdCl_2_ to give more scientific evidence on the protective role of gallic acid.

## Results

### Gallic acid enhanced antioxidant status in cadmium chloride-induced Wistar rats

**Figure 1:**
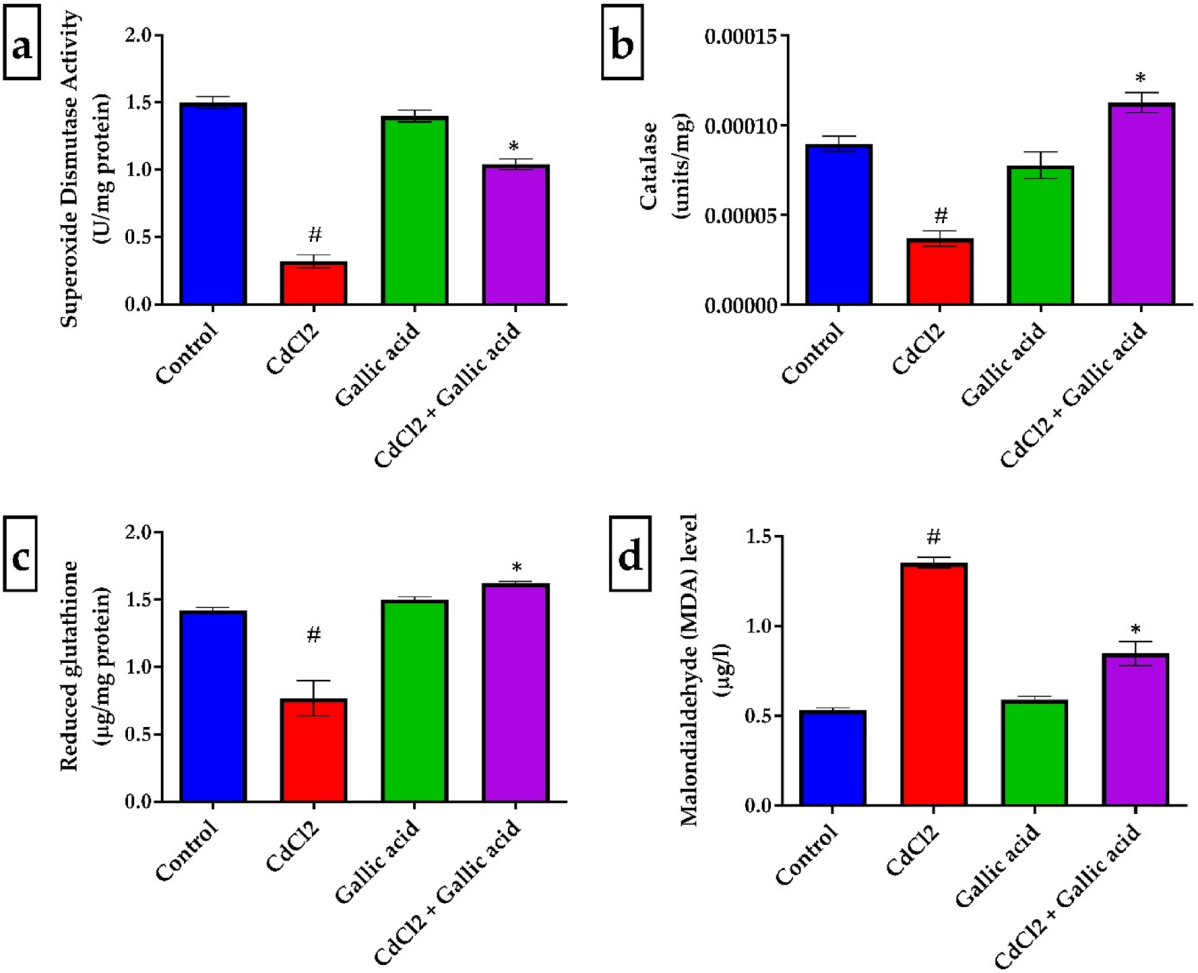
Brain cerebrum levels of antioxidants and MDA in control and treated groups. (**a**) SOD, (**b**) CAT, (**c**) GSH, and (**d**) MDA between the control and treated groups. The data is represented as mean ± SD (n = 5). ^#^Significant at *p* < 0.05 compared to the negative control; *Significant at *p* < 0.05 compared to the CdCl_2_ alone.

Figure 1 shows the effects of a 21-day exposure to CdCl_2_ and gallic acid on SOD, catalase, and GSH in the cerebrum of Wistar rats. CdCl_2_ alone was found to significantly (*p* < 0.05) reduce SOD, catalase, and GSH levels. On the other hand, CdCl_2_, markedly (*p* < 0.05) raised MDA levels in the cerebrum of experimental rats. However, compared to rats given only CdCl_2_, co-administration of CdCl_2_ and gallic acid at a dose of 20 mg/kg bwt markedly (*p* < 0.05) raised the activities of SOD and catalase as well as GSH level.

### Gallic acid reversed the inhibition of brain activities of acetylcholinesterase and butyrylcholinesterase in rats administered with CdCl_2_

**Figure 2:**
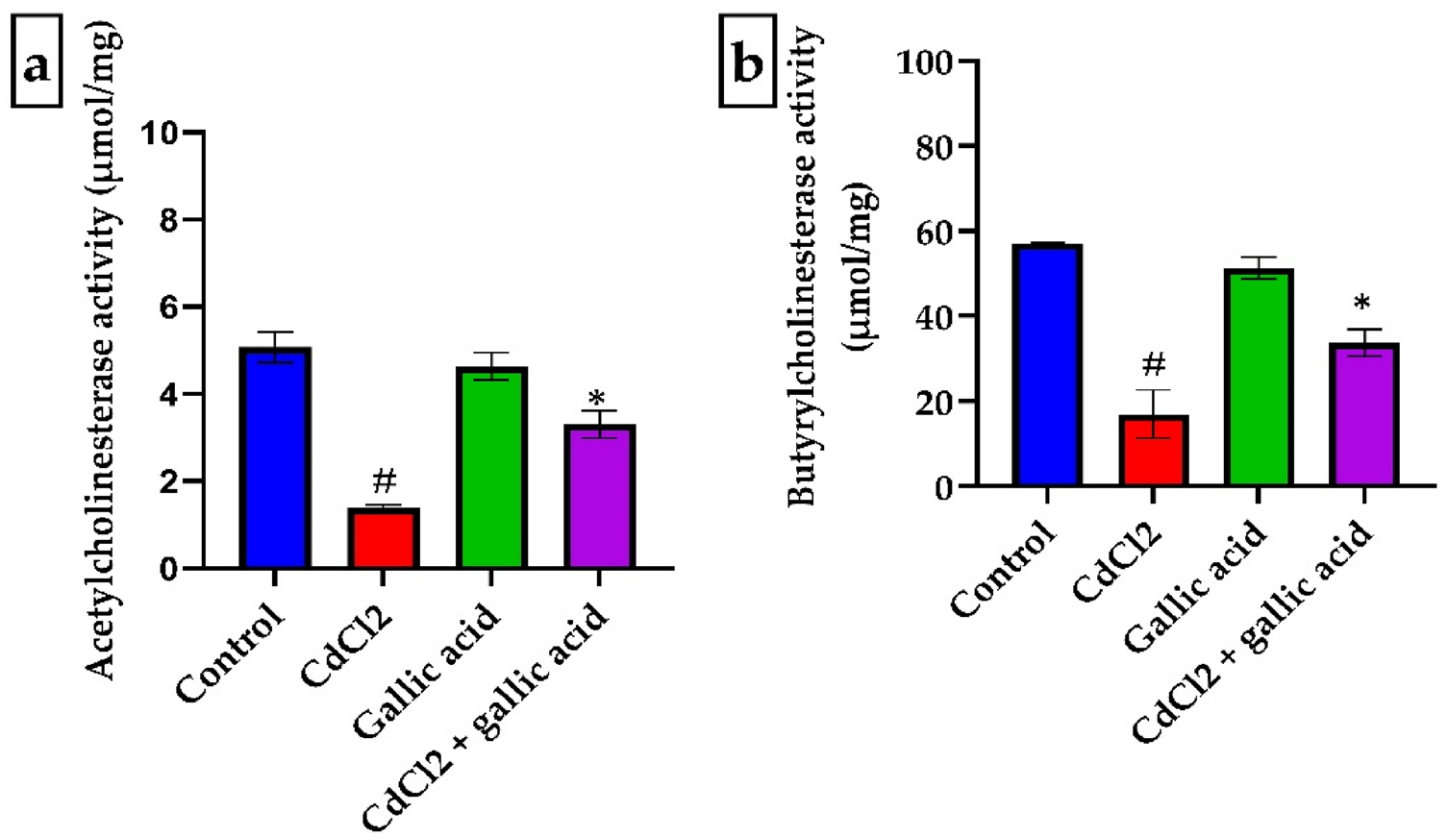
Gallic acid reverses the inhibition of (**a**) acetylcholinesterase (AChE) and (**b**) butyrylcholinesterase (BChE) of rats administered with distilled water, CdCl_2_ alone, gallic acid alone, and CdCl_2_ + gallic acid (20 mg/kg). The data is represented as mean ± SD (n = 5). ^#^Significant at p < 0.05 compared to the control; *Significant at *p* < 0.05 compared to the CdCl_2_ only group.

Figure 2 shows the impact of gallic acid on the brain activities of AChE and BChE in the cerebrum of rats given CdCl_2_. Compared to control rats, CdCl_2_ markedly (*p* < 0.05) inhibited AChE and BChE activities in the cerebrum of Wistar rats (Fig. 2). However, the activities of both enzymes were significantly decreased (*p* < 0.05) when CdCl_2_ and gallic acid were administered together.

### Gallic acid reverses brain levels of serotonin and dopamine in rats exposed to CdCl_2_

**Figure 3:**
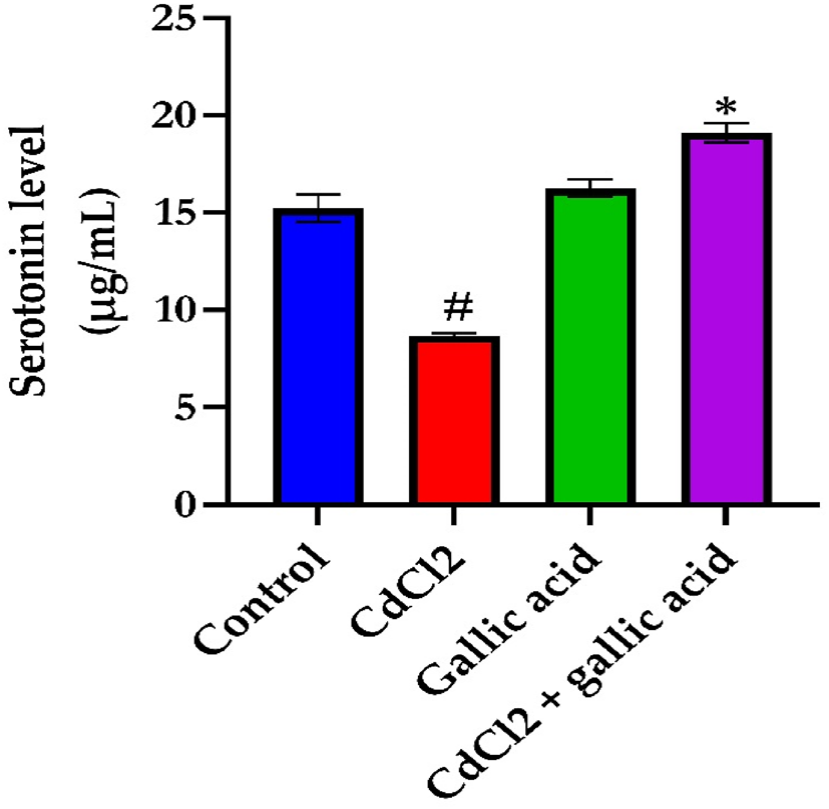
Gallic acid reverses the levels of serotonin in the cerebrum of rats administered with distilled water, CdCl_2_ alone, gallic acid alone, and CdCl_2_ + gallic acid (20 mg/kg). The data is represented as mean ± SD (n = 5). ^#^Significant at p < 0.05 compared to the control; *Significant at *p* < 0.05 compared to the CdCl_2_ only group.

**Figure 4:**
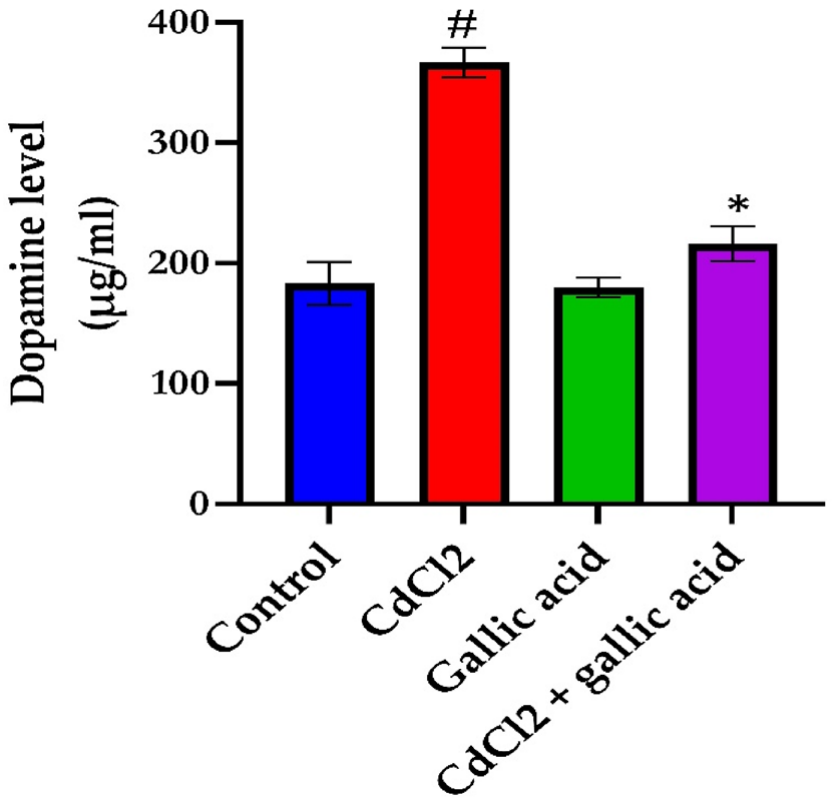
Gallic acid reverses the levels of dopamine in the cerebrum of rats administered with distilled water, CdCl_2_ alone, gallic acid alone, and CdCl_2_ + gallic acid (20 mg/kg). The data is represented as mean ± SD (n = 5). ^#^Significant at p < 0.05 compared to the control; *Significant at p < 0.05 compared to the CdCl_2_ only group.

The effect of gallic acid on brain neurotransmitter levels of serotonin (Fig. 3) and dopamine (Fig. 4) in the cerebrum of rats administered CdCl_2_. CdCl_2_ alone significantly (*p* < 0.05) reduced serotonin levels and increased dopamine levels in the brains of Wistar rats compared to control rats. On the contrary, the co-administration of gallic acid with CdCl_2_ significantly (*p* < 0.05) prevent such alterations in the level of neurotransmitters. Furthermore, in rats administered only gallic acid, serotonin levels increased and dopamine decreased compared to CdCl_2_ alone (*p* < 0.05).

### Gallic acid protected against CdCl_2_-induced neuro inflammation

**Figure 5:**
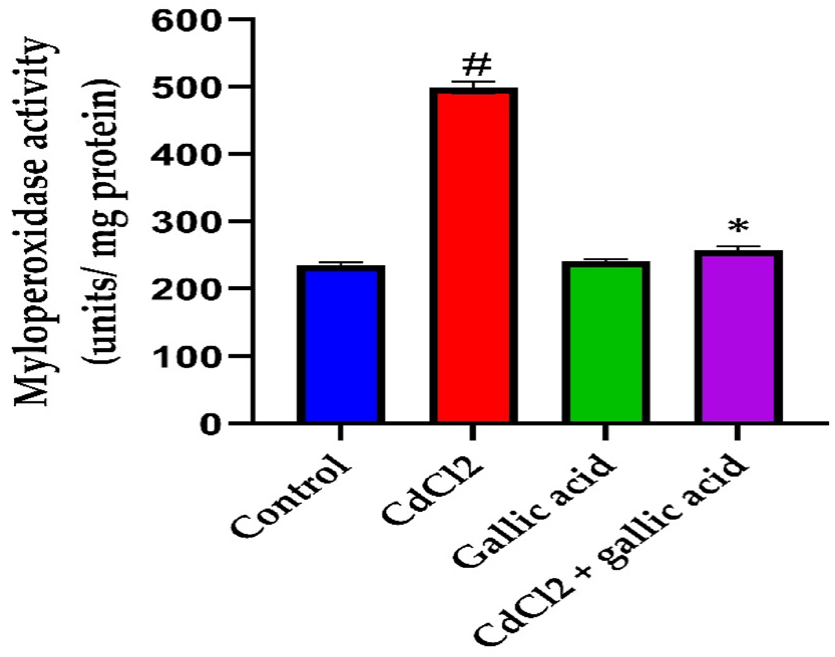
Gallic acid reverses brain cerebrum myeloperoxidase activity in CdCl_2_-induced neurotoxicity in Wistar rats. The data is represented as mean ± SD (n = 5). ^#^Significant at *p* < 0.05 compared to the control; *Significant at *p* < 0.05 compared to CdCl_2_ alone and CdCl_2_ + gallic acid.

**Figure 6:**
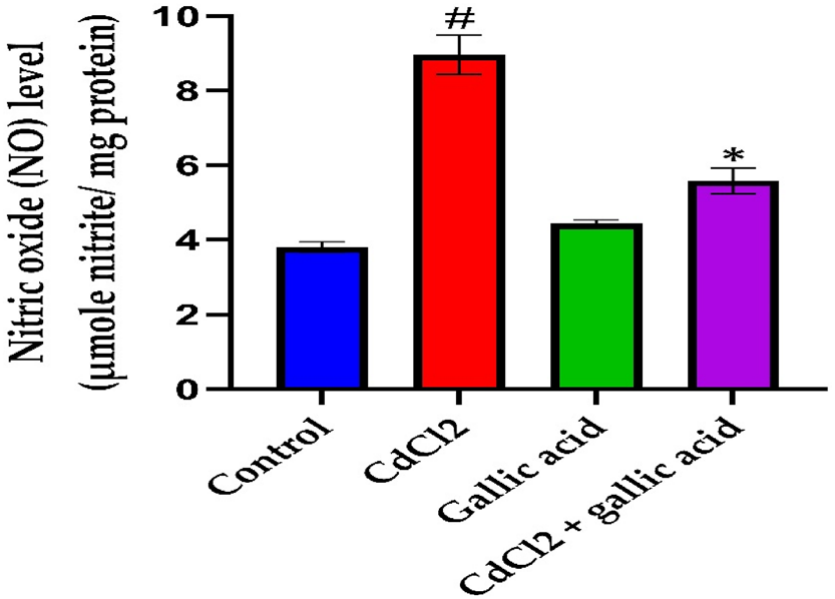
Impact of gallic acid on brain NO level in CdCl_2_-induced neurotoxicity in Wistar rats. The data is represented as mean ± SD (n = 5). ^#^Significant at *p* < 0.05 compared to the control; *Significant at *p* < 0.05 compared to CdCl_2_ alone group.

**Figure 7:**
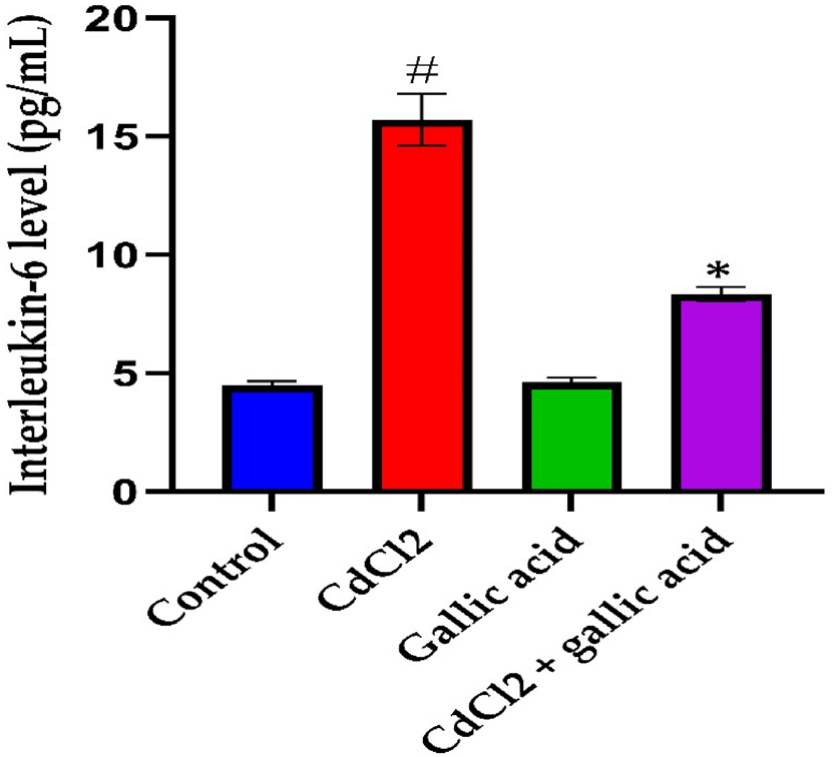
Impact of gallic acid on brain IL-6 level in CdCl_2_-induced neurotoxicity in Wistar rats. The data is represented as mean ± SD (n = 5). ^#^Significant at *p* < 0.05 compared to the control; *Significant at *p* < 0.05 compared to CdCl_2_ alone group.

The gallic acid effects on MPO, NO, and interleukin-6 (IL-6) brain activity in the cerebrum of rats receiving CdCl_2_ are shown in Figs. 5, 6, and 7. CdCl_2_ alone significantly (*p* < 0.05) raised the levels of MPO (Fig. 5), NO (Fig. 6), and IL-6 (Fig. 7) in comparison to the control. Co-administration of CdCl_2_ and gallic acid decreased NO and IL-6 levels as well as MPO activity in comparison to rats exposed to CdCl_2_ alone.

### Effect of gallic acid on membrane bound ATPases in rat brain

**Figure 8:**
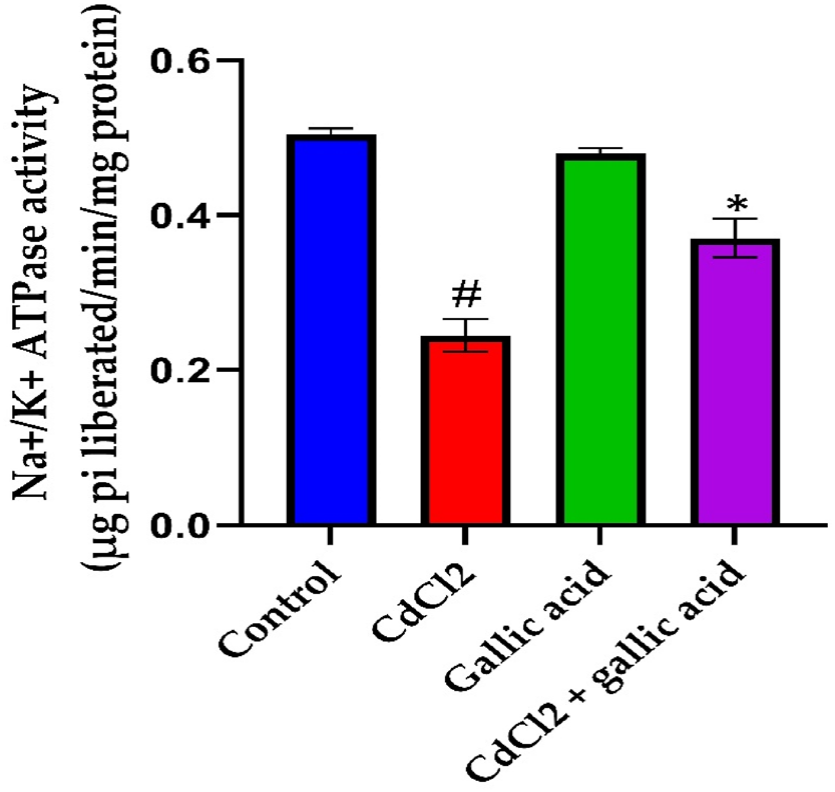
Impact of gallic acid and cadmium on the activities of membrane bound ATPases in the cerebrum of control and experimental rats. The data is represented as mean ± SD (n = 5). ^#^Significant at p < 0.05 compared to the control; *Significant at *p* < 0.05 compared to the CdCl_2_ only group.

Changes in ATPase enzyme activity (Na^+^/K^+^-ATPase) in the cerebrum of control and experimental rats are depicted in Fig. 8. Compared to control rats, CdCl_2_ alone rats had a significant decrease (*p* < 0.05) in the activity of the ATPase enzyme. Compared to cadmium alone, gallic acid co-administration significantly increased (*p* < 0.05) the activity of ATPase enzymes in the brain.

### Impact of gallic acid on brain of rats with CdCl_2_-induced neurotoxicity

**Figure 9:**
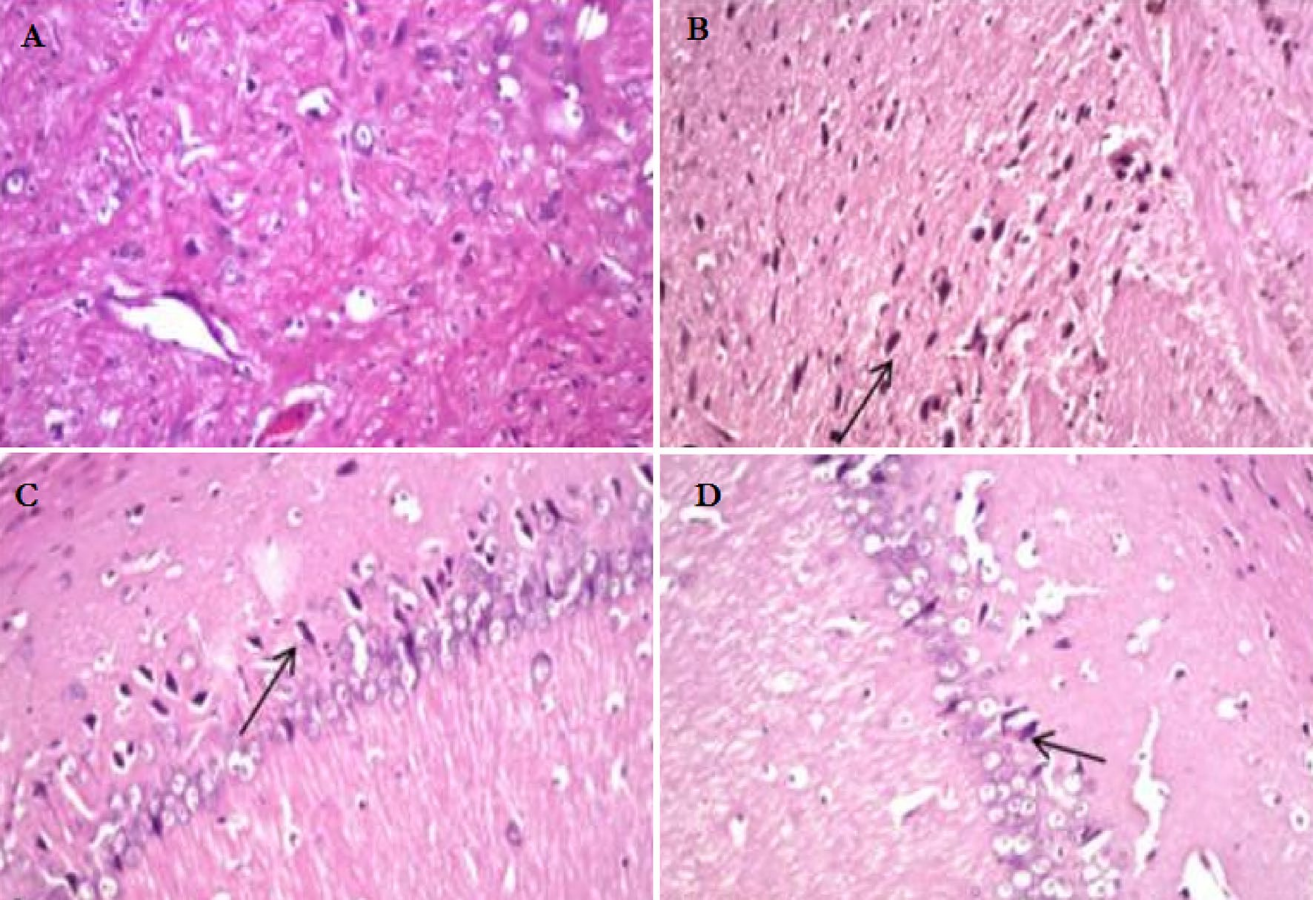
Impact of gallic acid on the cerebrum of CdCl_2_-induced neurotoxic rats (H & E, 200X). Group A (Control rats), Group B (cadmium chloride-treated rats), Group C (CdCl_2_ + GA-treated rats), Group D (gallic acid-treated rats) (**A**) Control showing normal granular layer, molecular layer, purkinje cell layer, blood vessels as well as while matter. (**B**) CdCl_2_ group showing severe vacuolation of purkinje cell layer, optical empty spaces due to necrosis (arrow), severe separation of purkinje cell layer from granular layer, severe hemorrhage of white matter; (**C**) CdCl_2_ + GA group showing vacuolated purkinje cell layer (black arrow), mild separation of purkinje cell layer; (**D**) GA group reveals normal granular layer, molecular layer, vacuolated purkinje cell layer (black arrow).

Figure 9 shows the histological studies of the cerebrum of experimental animals after the administration of CdCl_2_ + gallic acid. In contrast to the normal architecture displayed by the cerebrum of normal and gallic acid control rats, the cerebrum of CdCl_2_-induced rats had severe vacuolation of purkinje cell layer, optical empty spaces due to cell necrosis, severe separation of purkinje cell layer from granular layer, severe hemorrhage, and cell degeneration. The co-administration of CdCl_2_ with gallic acid decreased the occurrence of these changes in the cerebrum of rats and revealed near-normal architecture comparable to the CdCl_2_ alone rats.

## Discussion

According to recent research, Cd exposure in Wistar rats weakening antioxidant defense mechanisms, which messes up cellular redox and causes oxidative stress. During oxidative stress, free radicals are produced in large quantities, causing a reduction in antioxidant enzymes like superoxide dismutase and catalase^32^, which may have helped control ROS in the brain^33^. However, the brain can suffer harm if ROS is generated by interacting with the lipids, carbohydrates, proteins, and DNA present in the brain^31^. This study suggests that CdCl_2_ toxicity is caused by oxidative stress, which interacts with various brain cells and tissues. As a result, CdCl_2_-induced oxidative stress in rat brains, leading to decreased SOD and catalase activities, which is a sign of oxidative damage.

Shukla and Chandra^30^ and Ojo et al.^13,34^ found a similar decrease in catalase and SOD activities in rat brains after CdCl_2_ intoxication. The restoration of SOD and catalase activities in the brains of rats suggests that gallic acid has protective potential by reducing lipid peroxidation in brain tissues. Meanwhile, a drop in GSH levels has been noted in some earlier studies. Due to the excessive availability of the lipid peroxidation product (MDA) and inhibition of enzymes by CdCl_2_ metabolites, the level of GSH was lower in the current study. This could be due to its ability to conjugate electrophilic metabolites of CdCl_2_. Gallic acid, on the other hand, may help in the management of ROS in the brain as a neuroprotective agent due to its ability to prevent CdCl_2_-induced inhibition of GSH in the brain. The buildup of Cd in brain tissue, which depletes the GSH pool, may be the cause of the decrease in antioxidant enzyme levels observed in this study^35^. In addition to the binding of Cd to the sulfhydryl (-SH) group of the oxidative enzymes and inhibiting them, GSH depletion also renders the antioxidant enzymes inactive^36^. The current findings are consistent with those of Elkhadrag et al. and Ichipi Ifukor et al.^37,38^. Rutin, a well-known antioxidant, has been shown to have a similar effect on these antioxidant parameters^39^.

The neurotoxin potency of Cd has been demonstrated in both in vitro and in vivo studies^40,41^. Animals exposed to Cd have been found to have altered catecholaminergic and serotoninergic transmission^42,43^. To determine the neurotoxic effects of heavy metals, it is imperative to investigate the activities of brain enzymes like AChE and BChE. According to several studies^44^, AChE activity in the brain is believed to decline as a result of free radical production in the brain. Acetylcholine accumulation, which results in cholinergic hyperactivity, convulsions, and epileptic status, has been shown to be caused by decreased AChE^45^. Although it is unclear how much it directly contributes, decreased levels of AChE in the brain could be one of the indicators of Cd-induced injury because they are a key regulator of behavioral processes. The fact that CdCl_2_ inhibited the activities of butyrylcholinesterase and acetylcholinesterase in the brains of rats in this study indicates that acetylcholine had accumulated due to disruptions cholinergic transmission. This result is consistent with Pari and Murugavel's report on the effect of diallyl tetrasulfide, which is commonly found in garlic, on AChE^46^.

The most common neurotransmitter in the central nervous system, serotonin (5-HT), is essential for memory and learning^47^. On the other hand, tryptophan hydroxylase is distributed similarly to serotonin (5-HT) in the hypothalamus, the midbrain, and the limbic system of the brain^48–50^. In this study, rats treated with cadmium alone had considerably lower levels of 5-HT in their brains. By inhibiting the oxidative metabolism of serotonergic neurons, cadmium prevents tryptophan hydroxylase activity, which is essential for the conversion of tryptophan to 5-HT^51^. Our results are in line with those of Das et al.^52^, who discovered that rats given CdCl_2_ had markedly lower levels of 5-HT in their brains. In the current study, brain catecholamine levels (dopamine) increased in rats administered cadmium. It has been proposed that it causes changes in neurochemistry as a result of oxidative damage to brain tissue, which may be responsible for its toxic action^53,54^. Cd also promotes lipid peroxidation by increasing the production of superoxide anions^55^, which act as primary oxidant. This process can cause direct cell damage by destroying membranes and indirect cell damage by producing reactive carbonyl products^56^. Cd-induced lipid peroxidation is the cause of membrane damage that leads to neuronal dysfunction, resulting in decreased dopamine uptake in the brain^57^. Our findings are consistent with^30^, who found that low-dose chronic cadmium treatment markedly increased dopamine levels in rats. Additionally^58^, found that low-dose chronic cadmium treatment elevated dopamine levels in rats.

Due to the presence of free fatty acids in the brain, it is extremely prone to lipid peroxidation^59^. The increase in MDA levels caused by CdCl_2_ in the current study suggests that the brains of rats may experience cellular damage and dysfunction as a result of excessive ROS generation^60^. This supports the hypothesis that a decrease in glutathione levels is the first sign of oxidative stress. Rotimi et al.^14^ made a similar finding of elevated MDA levels in CdCl_2_-induced rats. On the other hand, gallic acid's capacity to restore MDA levels that are comparable to those of the relevant controls may be connected to its anti-peroxidative properties.

One of the defenders against neurotoxic assaults on a host's nervous system is neuroinflammation. As a promising clinical target, neuroinflammation is also a frequent pathological outcome or etiology of neurodegeneration and neurological disorders^61^. Therefore, the detection of specific neuroinflammation markers was crucial in this study. For example, the pathogenesis of inflammatory disorders and the maintenance of neural tissue homeostasis depend on the cytokine interleukin-6 (IL-6)^62^. Nitric oxide enhances cerebral blood flow while acting as a retrogressive neurotransmitter in synapses and is crucial for intracellular signaling in neurons^63^. Myeloperoxidase is a therapeutic target for oxidative stress and neuroinflammation-causing pathological processes^64^. The rise in IL-6 levels in the brains of rats suggests that it interferes with the physiological processes associated with neuroinflammation. This justify the role of IL-6 in the pathogenesis of neurodegenerative diseases. In this study, nitric oxide levels were higher than normal in the brains of rats due to CdCl_2_, which may also have interfered with intracellular signaling in the brain. Reduced antioxidant status with elevated oxidative stress was associated with increased MPO activity in the rat brain. Gallic acid, on the other hand, has the potential to be an anti-inflammatory agent in inflammation-induced brain diseases, as evidenced by its ability to reverse the increase in NO and MPO induced by CdCl_2_.

The production of metabolic energy, the uptake and release of catecholamines and serotonin, as well as neural excitability, are all regulated by the enzyme Na^+^/K^+^-ATPase^65,66^. Furthermore, Mg^2+^ -ATPase is responsible for keeping intracellular Mg^2+^ levels in the brain at a high level, where changes can affect the rate of protein synthesis and cell growth^67^. In the nervous system, Ca^2+^ acts as a second messenger. Numerous pathological lesions in the brain are caused by variations in Ca^2+^ levels^68^ . The Ca2 + ATPase activity in the cell membrane is also inhibited by the Ca^2+^ overload caused by Cd, which ultimately results in irreversible cell death. Exposure to Cd affects the activities of these ATPase enzymes^17,43,69,70^, indicating changes in membrane and neurotransmitter functions. Compared to control rats, CdCl_2_ alone showed a significant decrease in ATPase enzyme activity in the brains of experimental animals. ATPase activity can be reduced due to the formation of Cd-ATPase complexes through the enzyme’s SH group and/or increased oxidative stress^46^. Co-administration of gallic acid with Cd significantly increased the activity of ATPase enzymes in the brain compared to cadmium alone.

Significant histopathological changes in brain tissue have been widely associated with heavy metals^71^. According to histological examination of brain tissue, Cd intoxication causes abnormal structural changes in brain tissue, including apoptosis, nuclear vacuolization, and inflammatory lymphocytic changes. Cd alters the cortical micro-and macrostructures once it enters the brain. Cd destroys glial and neuronal cells in the hippocampus’s white matter^72^. Cadmium causes severe damage to the brain, including encephalopathy and hemorrhage. These changes are neuropathological and neurochemical. The cortical neuroglia, pyramidal, and granule cells are also altered by Cd exposure^73^. Additionally, Cd affects the structure of nerve cells and parenchyma, impairing attention, memory, and olfactory functions as well as hypernociception^74^. According to a histopathological assessment of the cerebrum, the intoxication with cadmium led to abnormal ultra-structural changes, including severe vacuolation of purkinje cell layer, optical empty spaces due to cell necrosis, severe separation of purkinje cell layer from granular layer, severe hemorrhage, and cell degeneration. The observed pathological damage caused by CdCl_2_-induced rats treated with gallic acid showed a significant recovery, indicating that gallic acid is capable of abating the neuronal impairment caused by cadmium. As a result, it might be hypothesized that gallic acid could prevent brain damage caused by Cd.

Our obtained data showed that CdCl_2_ induced oxidative stress and neuroinflammation in the brains of Wistar rats. However, gallic acid offered neuroprotective effects on CdCl_2_-induced toxicity in the brain, indicating its antioxidant and therapeutic potentials against oxidative stress and neuro-inflammation in the brain of rats exposed to CdCl_2_. Our results suggested that gallic acid showed a profound improvement in neurotransmitter levels.

## Materials and methods

### Chemicals

Gallic acid (GA) was a product of SantaCruz Biotechnology, Heidelberg, Germany. ELISA kits for serotonin, dopamine, and interleukin-6 were purchased from Elabscience, Houston, Texas, USA. All other chemicals were of analytical grade.

### Animals

For this experiment, twenty (20) Wistar rats weighing between 170 and 200 g were used. The animals were acquired from Landmark University's Biochemistry Department in Kwara State, Nigeria. Throughout the 21-day acclimatization period and the experimental period, they were kept at room temperature with unlimited access to food and water (21 days).

### Ethical approval

All experimental rats used in this study were handled in accordance with the rules and regulations established for animal management in research, as outlined in NIH Publications No. 80-23 revised, 1996. The Animal Care and Use Ethical Committee at Landmark University, Nigeria (LUAC/2020/0052B) confirmed and approved that the experimental treatment of the rats is in accordance with ARRIVE guidelines. Furthermore, all methods/experimental protocol/s were approved by the institution's ethical committee and ARRIVE guidelines.

### Animal groupings and experimental procedures

The rats were divided into four groups (n = 5) at random. The negative control group was given distilled water only for the duration of the study. Group 2 was the positive control, receiving only CdCl_2_ (5 mg/kg/day) throughout the study. Group 3 received only 20 mg/kg of gallic acid per day, whereas Group 4 received 20 mg/kg of gallic acid + 5 mg/kg of CdCl_2_. The doses of cadmium and gallic acid were chosen based on previous studies^6,75^ for cadmium and^13,76^ for gallic acid. Gallic acid was said to be safe at a daily dose of 20 mg/kg body weight^76^, but it has been demonstrated that the cadmium dose chosen causes significant oxidative stress in various tissues^34,77^.

### Serum and organ collection

Animals were sacrificed under halothane anesthesia by cervical dislocation twenty-four hours after the last administration. Cardiovascular puncture was used to quickly collect blood into a plain bottle, which was then spun at 3000 rpm for 10 min to obtain serum, which was used to assess biochemical indices. Quickly removed from the body, the brain was rinsed in ice-cold normal saline and the cerebrum was homogenized in 0.1 M phosphate buffer (1:10 w/v) (pH 7.4). The brains were sectioned and the cerebrum were separated. At 3000 rpm, the homogenates were centrifuged. The obtained clear supernatant was used for various biochemical assays.

### Biochemical analyses

Using Ellman's method^78^, the activities of acetylcholinesterase (AChE) and butyrylcholinesterase were assessed. The TBA Reactive Substances (TBARS) concentration was measured using the Buege and Aust method^79^. The Na+/K+ ATPase's activity was assessed by Bonting^80^. The technique outlined by Misra and Fridovich^81^ was used to test the superoxide dismutase activity. The catalase activity was tested using the procedure outlined by Aebi^82^. The method outlined by Ellman^83^ was used to measure the level of reduced glutathione (GSH). The assay for myeloperoxidase activity was carried out using the technique described by Granell et al.^84^. The Griess reaction was used to calculate the nitric oxide level^85^. The levels of IL-6, dopamine, and serotonin were estimated using rat specific enzyme linked immunosorbent assay (ELISA) kits (ElabScience, USA) according to the manufacturer’s instructions.

### Histopathological investigation

Animal brain tissues were used for histopathological research. The tissues underwent routine processing before being formalin-fixed in 10% formalin and embedded in paraffin wax. Hematoxylin and eosin (H and E) were used to stain the cerebrum sections, which were of 4 µm thick, on glass slides. A light microscope was used to examine the slides, and magnified images (200X) of the tissue structures were recorded^86^.

### Data analysis

Data analysis was performed using one-way analysis of variance (ANOVA) on GraphPad Prism 9.0, version (GraphPad software, Inc, San Diego, USA). All results were expressed as mean ± standard deviation (SD). Statistical significance at p < 0.05 was determined by using Tukey's post hoc multiple comparisons test.

## Data Availability

Data is available on reasonable request from the corresponding author.

## References

[1] ATSDR (Agency for Toxic Substances and Disease Registry) (2005). U.S. Toxicological Profile for Cadmium.

[2] Ali H, Khan E, Ialhi I (2019). Environmental chemistry and ecotoxicology of hazardous heavy metals: Environmental persistence, toxicity, and bioaccumulation. J. Chem..

[3] Genchi G, Sinicropi MS, Lauria G, Carocci A, Catalano A (2020). The effects of cadmium toxicity. Int. J. Environ. Res. Public Health.

[4] Satarug S, Garrett SH, Sens MA, Sens DA (2010). Cadmium, environmental exposure and health outcomes. Environ. Health Perspect..

[5] Renugadevi J, Miltonprabu S (2009). Protective role of alpha tocopherol and ascorbic acid against cadmium induced neurotoxicity in rats. Int. J. Med. Sci..

[6] El-demerdash FM, Yousef MI, Kedwany FS, Baghdadi HH (2004). Cadmium-induced changes in lipid peroxidation, blood hematology, biochemical parameters and semen quality of male rats: Protective role of vitamin E and beta-carotene. Food Chem. Toxicol..

[7] González-Trujano E, Navarrete A (2011). Effect of zinc on the cadmium acute intoxication in the gastric injury induced in rats. Rev. Latinoamer. Quím.

[8] Branca JJ, Morucci G, Pacini A (2018). Cadmium-induced neurotoxicity: Still much ado. Neural Regen. Res..

[9] Limón-Pacheco J, Gonsebatt ME (2009). The role of antioxidants and antioxidant-related enzymes in protective responses to environmentally induced oxidative stress. Mutation Res..

[10] Ajiboye BO (2018). Inhibitory effect of ethyl acetate fraction of *Solanum macrocarpon* L. leaves on cholinergic, monoaminergic and purinergic enzyme activities. Food Biochem..

[11] Ajiboye BO, Ojo OA, Okesola MA, Oyinloye BE, Kappo AP (2018). Ethyl acetate leaf fraction of *Cnidoscolus aconitifolius* (Mill.) I. M. Johnst: Antioxidant potential, inhibitory activities of key enzymes on carbohydrate metabolism, cholinergic, monoaminergic, purinergic and chemical fingerprinting. Int. J. Food Crops.

[12] Oboh G, Adebayo AA, Ademosun AO (2019). Rutin alleviates cadmium-induced neurotoxicity in Wistar rats: Involvement of modulation of nucleotide-degrading enzymes and monoamine oxidase. Metab. Brain Dis..

[13] Shukla GS, Chandra SV (1987). Concurrent exposure to lead, manganese, and cadmium and their distribution to various brain regions, liver, kidney, and testis of growing rats. Arch. Environ. Contam. Toxicol..

[14] Chen L, Xu B, Liu L, Luo Y, Zhou H, Chen W (2011). Cadmium induction of reactive oxygen species activates the mTOR pathway, leading to neuronal cell death. Free Radic. Biol. Med..

[15] Ojo OA, Rotimi D, Emmanuel B, Kayode OT, Ojo AB, Alejolowo O, Ajiboye BO, Oluba OM (2021). Gallic acid protects against cadmium chloride-induced alterations in Wistar rats via the antioxidant defense mechanism. J. Pharm. Pharmacogn. Res.

[16] Rotimi D, Ojo OA, Emmanuel B, Ojo AB, Elebiyo TC, Nwonuma CO, Oluba OM (2021). Protective impacts of gallic acid against cadmium-induced oxidative toxicity in the ovary of rats. Comp. Clin. Pathol..

[17] EL-missiry MA, Shalaby F (2000). Role of beta-carotene in ameliorating the cadmium-induced oxidative stress in rat brain and testis. J. Biochem. Mol. Toxicol..

[18] Nayeem N, Asdaq SMB, Salem H, Ahel-Alfqy S (2016). Gallic acid: A promising lead molecule for drug development. J. Appl. Pharm..

[19] Kahkeshani N, Farzaei F, Fotouhi M, Alavi SS, Bahramsoltani R, Naseri R, Bishayee A (2019). Pharmacological effects of gallic acid in health and disease: A mechanistic review. Iran J. Basic Med. Sci..

[20] Gao J, Hu J, Hu D, Yang X (2019). A role of gallic acid in oxidative damage diseases. Nat. Prod. Commun..

[21] Wu XC, Yu BT, Hou AL, Hu TT, Lu GQ (2006). Study on stability of gallic acid. Med. J. Natl. Def. Forces Southwest China.

[22] Mansouri MT, Farbood Y, Sameri MJ, Sarkaki A, Naghizadeh B, Rafeirad M (2013). Neuroprotective effects of oral gallic acid against oxidative stress induced by 6-hydroxydopamine in rats. Food Chem..

[23] Sarkaki A, Farbood Y, Gharib-Naseri MK, Badavi M, Mansouri MT, Haghparast A (2015). Gallic acid improved behavior, brain electrophysiology, and inflammation in a rat model of traumatic brain injury. Can. J. Physiol. Pharmacol..

[24] Inoue M (1995). Selective induction of cell death in cancer cells by gallic acid. Biol. Pharm. Bull..

[25] Ohno Y, Fukuda K, Takemura G, Toyota M, Watanabe M, Yasuda N, Xinbin Q, Maruyama R, Akao S, Gotou K (1999). Induction of apoptosis by gallic acid in lung cancer cells. Anticancer Drugs.

[26] Priscilla DH, Prince PSM (2009). Cardioprotective effect of gallic acid on cardiac troponin-T, cardiac marker enzymes, lipid peroxidation products and antioxidants in experimentally induced myocardial infarction in Wistar rats. Chem. Biol. Interact..

[27] Patel SS, Goyal RK (2011). Cardioprotective effects of gallic acid in diabetes-induced myocardial dysfunction in rats. Pharmacogn. Res..

[28] Rasool MK, Sabina EP, Ramya SR, Preety P (2010). Hepatoprotective and antioxidant effects of gallic acid in paracetamol-induced liver damage in mice. J. Pharm. Pharmacol..

[29] Ohno T, Inoue M, Ogihara Y (2001). Cytotoxic activity of gallic acid against liver metastasis of mastocytoma cells P-815. Anticancer. Res..

[30] Kim MJ, Seong AR, Yoo JY (2011). Gallic acid, a histone acetyltransferase inhibitor, suppresses β-amyloid neurotoxicity by inhibiting microglial-mediated neuroinflammation. Mol. Nutr Food Res..

[31] Kroes BV, Van den Berg A, Van Ufford HQ, Van Dijk H, Labadie R (1992). Anti-inflammatory activity of gallic acid. Planta Med..

[32] Birben E, Sahiner UM, Sackesen C, Erzurum S, Kalayci O (2012). Oxidative stress and antioxidant defense. World Allergy Organ. J..

[33] Kaygusuzoglu E, Caglayan C, Kandemir FM, Yıldırım S, Kucukler S, Kılınc MA, Saglam YS (2018). Zingerone ameliorates cisplatin-induced ovarian and uterine toxicity via suppression of sex hormone imbalances, oxidative stress, inflammation and apoptosis in femalewistar rats. Biomed. Pharmacother..

[34] Ojo OA, Oyinloye BE, Ajiboye BO, Onikanni SA (2014). Neuroprotective mechanism of ethanolic extract of *Irvingia gabonensis* stem bark against cadmium-induced neurotoxicity in rats. Br. J. Med. Med. Res..

[35] Onyema OO, Farombi EO, Emerole GO, Ukoha AI, Onyeze GO (2006). Effect of vitamin E on monosodium glutamate induced hepatotoxicity and oxidative stress in rats. Indian J. Biochem. Biophys..

[36] Renugadevi J, Prabu SM (2009). Naringenin protects against cadmium-induced oxidative renal dysfunction in rats. Toxicology.

[37] Elkhadragy MF, Al-Olayan EM, Al-Amiery AA, Abdel Moneim AE (2018). Protective effects of *Fragaria ananassa* extract against cadmium chloride-induced acute renal toxicity in rats. Biol. Trace Elem. Res..

[38] Ichipi-Ifukor PC, Asagba SO, Nwose C, Mordi JC, Oyem JC (2022). Palm oil extracts protected against cadmium chloride poisoning via inhibition of oxidative stress in rats. Bull. Natl. Res. Cent..

[39] Mostafa DG, Khaleel EF, Badi RM, Abdel-Aleem GA, Abdeen HM (2019). Rutin hydrate inhibits apoptosis in the brains of cadmium chloride-treated rats via preserving the mitochondrial integrity and inhibiting endoplasmic reticulum stress. Neurol. Res..

[40] Webster WS, Valois AA (1981). The toxic effect of cadmium on the neonatal mouse CNS. J. Neuropathol. Exp. Neurol..

[41] Kabeer IA, Rajender RJ, Desaiah D (1989). Protection against cadmium toxicity and enzyme inhibition by dithiothreitol. Cell Biochem. Funct..

[42] Gupta A, Gupta A, Shukla SG (1993). Neurochemical changes in developing rat brain after pre- and postnatal cadmium exposure. Bull. Environ. Contam. Toxicol..

[43] Antonio MT, Corredor L, Leret ML (2003). Study of the activity of several brain enzymes like markers of the neurotoxicity induced by perinatal exposure to lead and/or cadmium. Toxicol. Lett..

[44] Tsakiris S, Angelogianni P, Schulpis KH, Stavridis JC (2000). Protective effect of l-phenylalanine on rat brain acetylcholinesterase inhibition induced by free radicals. Clin. Biochem..

[45] Olney JW, Collins RC, Sloviter RS (1986). Exotoxic mechanisms of epileptic brain damage. Adv. Neurol..

[46] Pari L, Murugavel P (2007). Diallyl tetrasulfide improves cadmium induced alterations of acetylcholinesterase, ATPases and oxidative stress in brain of rats. Toxicology.

[47] Butzlaff M, Ponimaskin E, Butzlaff M, Ponimaskin E (2016). The role of serotonin receptors in Alzheimer's disease. Opera Med. Physiol..

[48] Ney DM, Murali SG, Stroup BM, Nair N, Sawin EA, Rohr F, Levy HL (2017). Metabolomic changes demonstrate reduced bioavailability of tyrosine and altered metabolism of tryptophan via the kynurenine pathway with ingestion of medical foods in phenylketonuria. Mol. Genet. Metab..

[49] Fernstrom JD, Fernstrom MH (2007). Tyrosine, phenylalanine, and catechol- amine synthesis and function in the brain. J. Nutr..

[50] Haider S, Khaliq S, Ahmed SP, Haleem DJ (2006). Long-term tryptophan administration enhances cognitive performance and increases 5HT metabolism in the hippocampus of female rats. Amino Acids.

[51] Batool Z, Agha F, Ahmad S, Liaquat L, Tabassum S, Khaliq S, Anis L, Sajid I, Emad S, Perveen T, Haider S (2017). Attenuation of cadmium-induced decline in spatial, habituation and recognition memory by long-term administration of almond and walnut supplementation: Role of cholinergic function. Pak. J. Pharm. Sci..

[52] Das K, Das P, Dasgupta S, Dey C (1993). Serotonergic-cholinergic neurotransrnitters' functionin brain during cadmium exposure in proteinrestricted rat. Biol. Trace Elem. Res..

[53] Wang B, Du Y (2013). Cadmium and its neurotoxic effects. Oxid. Med. Cell Longev..

[54] Wang H, Zhang L, Abel GM, Storm DR, Xia Z (2018). Cadmium exposure impairs cognition and olfactory memory in male C57BL/6 mice. Toxicol. Sci..

[55] Wright RO, Baccarelli A (2007). Metals and neurotoxicology. J Nutr.

[56] Esterbauer, H., Zollner, H. & Schaur, R. In *Lipidoxidation* (Vigo, C., Pel Frey, B. A., eds) 239–283 (CRC Press, 1990).

[57] Rajanna B, Hobson M, Boykin M, Chetty C (1990). Effects of chronic treatment with cadmium on ATPases, uptake of catecholamines and lipid peroxidation in rat brain synaptosomes. Ecotoxicol. Environ. Saf..

[58] Zhai Q, Narbad A, Chen W (2015). Dietary strategies for the treatment of cadmium and lead toxicity. Nutrients.

[59] Yan HF, Zou T, Tuo QZ, Xu S, Li H, Belaidi AA, Lei P (2021). Ferroptosis: Mechanisms and links with dieases. Signal Transduct. Target. Ther..

[60] Ajayi AM, Ben-Azu B, Godson JC, Umukoro S (2020). Effect of spondias mombin fruit extract on scopolamine-induced memory impairment and oxidative stress in mice brain. J. Herbs. Spices Med. Plants.

[61] Mishra A, et al. (2021) Neuroinflammation in Neurological Disorders: Pharmacotherapeutic Targets from Bench to Bedside. Springer, Berlin 10.1007/s11011-021-00806-4 34387831

[62] Rothaug M, Becker-Pauly C, Rose-John S (2016). The role of interleukin-6 signaling in nervous tissue. Biochim. Biophys. Acta.

[63] Picón-Pagès P, Garcia-Buendia J, Muñoz FJ (2019). Functions and dysfunctions of nitric oxide in brain. Biochim. Biophys. Acta.

[64] Chen S, Chen H, Du Q, Shen J (2020). Targeting myeloperoxidase (MPO) mediated oxidative stress and inflammation for reducing brain ischemia injury: Potential application of natural compounds. Front. Physiol..

[65] Bogdanski DF, Tissuri A, Brodie BB (1968). Role of sodium, potassium, ouabain and reserpine in uptake, storage and metabolism of biogenic amines in synaptosomes. Life Sci..

[66] Mata M, Fink DJ, Gainer H, Smith CB, Davidsen L, Savakis H, Schwartz WJ, Sokoloff L (1980). Activity-dependent energy metabolism in rat posterior pituitary, primarily reflects sodium pump activity. J. Neurochem..

[67] Sanui H, Rubin H, Boynton AL, McKochan WL, Whitfield JP (1982). The role of magnesium in cell proliferation and transformation. Ions, Cell Proliferation and Cancer.

[68] Repetto M (1997). Toxicologia Fundamental.

[69] Rajanna B, Hobson M, Boykin M, Chetty CS (1990). Effects of chronic treatment with cadmium on ATPases, uptake of catecholamines and lipid peroxidation in rat brain synaptosomes. Ecotoxicol. Environ. Saf..

[70] Carfagna MA, Ponsler GD, Muhoberac BB (1996). Inhibition of ATPase activity in rat synaptic plasma membranes by simultaneous exposure to metals. Chem. Biol. Interact..

[71] Al-Quraishy S, Dkhil MA, Ibrahim SR, Abdel Moneim AE (2016). Neuroprotective potential of Indigofera oblongifolia leaf methanolic extract against lead acetate-induced neurotoxicity. Neural Regen. Res..

[72] El-Tarras AES, Attia HF, Soliman MM, El Awady MA, Amin AA (2016). Neuroprotective effect of grape seed extract against cadmium toxicity in male albino rats. Int. J. Immunopathol. Pharmacol..

[73] Afifi OK, Embaby AS (2016). Histological study on the protective role of ascorbic acid on cadmium induced cerebral cortical neurotoxicity in adult male albino rats. J. Microsc. Ultrastruct..

[74] Wang B, Du Y (2013). Cadmium and its neurotoxic effects. Oxid. Med. Cell. Longev..

[75] Shagirtha K, Muthumani M, Milton Prabu S (2011). Melatonin abrogates cadmium induced oxidative stress related neurotoxicity in rats. Euro Rev. Med. Pharmacol. Sci..

[76] Ola-Davies OE, Olukole SG (2018). Gallic acid protects against bisphenol A-induced alterations in the cardiorenal system of Wistar rats through the antioxidant defense mechanism. Biomed. Pharmacother..

[77] Ojo OA, Ajiboye BO, Oyinloye BE, Ojo AB, Olarewaju OI (2014). Protective effect of *Irvingia gabonensis* stem bark extract on cadmium-induced nephrotoxicity in rats. Interdiscip. Toxicol..

[78] Ellman GL, Courtney KD, Andres V, Featherstone RM (1961). A new and rapid colorimetric determination of acetylcholinesterase activity. Biochem. Pharmacol..

[79] Buege JA, Aust SD (1978). Biomembranes-Part C: Biological oxidations. Methods Enzymol..

[80] Bonting SL, Bilter EE (1970). Presence of enzyme system in mammalian tissues. Membrane and Ion Transport.

[81] Misra HP, Fridovich I (1972). The role of superoxide anion in the autooxidation of epinephrine and a simple assay for superoxide dismutase. J. Biol. Chem..

[82] Aebi H, Bilter EE (1974). Catalase estimation. Methods of Enzymatic Analysis.

[83] Ellman GL (1959). Tissue sulfhydryl groups. Arch. Biochem. Biophys..

[84] Granell S, Gironella M, Bulbena O, Panés J, Mauri M, Sabater L, Aparisi L, Gelpi E, Closa D (2003). Heparin mobilizes xanthine oxidase and induces lung inflammation in acute pancreatitis. Crit. Care Med..

[85] Green LC, Wagner DA, Glogowski J, Skipper PL, Wishnok JS, Tannenbaum SR (1982). Analysis of nitrate, nitrite, and [15N]nitrate in biological fluids. Anal. Biochem..

[86] Drury RA, Wallington EA, Cancerson R (1976). Carlton's Histopathological Techniques.

